# Supplementary material for: Implementation of an Electronic National Early Warning System to Decrease Clinical Deterioration in Hospitalized Patients at a Tertiary Medical Center
Source: Int J Environ Res Public Health. 2021 Apr 25;18(9):4550. doi: 10.3390/ijerph18094550 (PMC8123282; doi:10.3390/ijerph18094550)
Supplement: Supplementary file 1 [file ijerph-18-04550-s001.zip › ijerph-1171383-supplementary.pdf]

Supplementary file

Supplementary Figure 1. The score of E-NEWS could be drilled down to view the original data, including respiratory rate, consciousness, systolic blood pressure, O2 therapy, pulse rate, and sPO2. The trend of each item can be traced from the time that a patient is admitted.

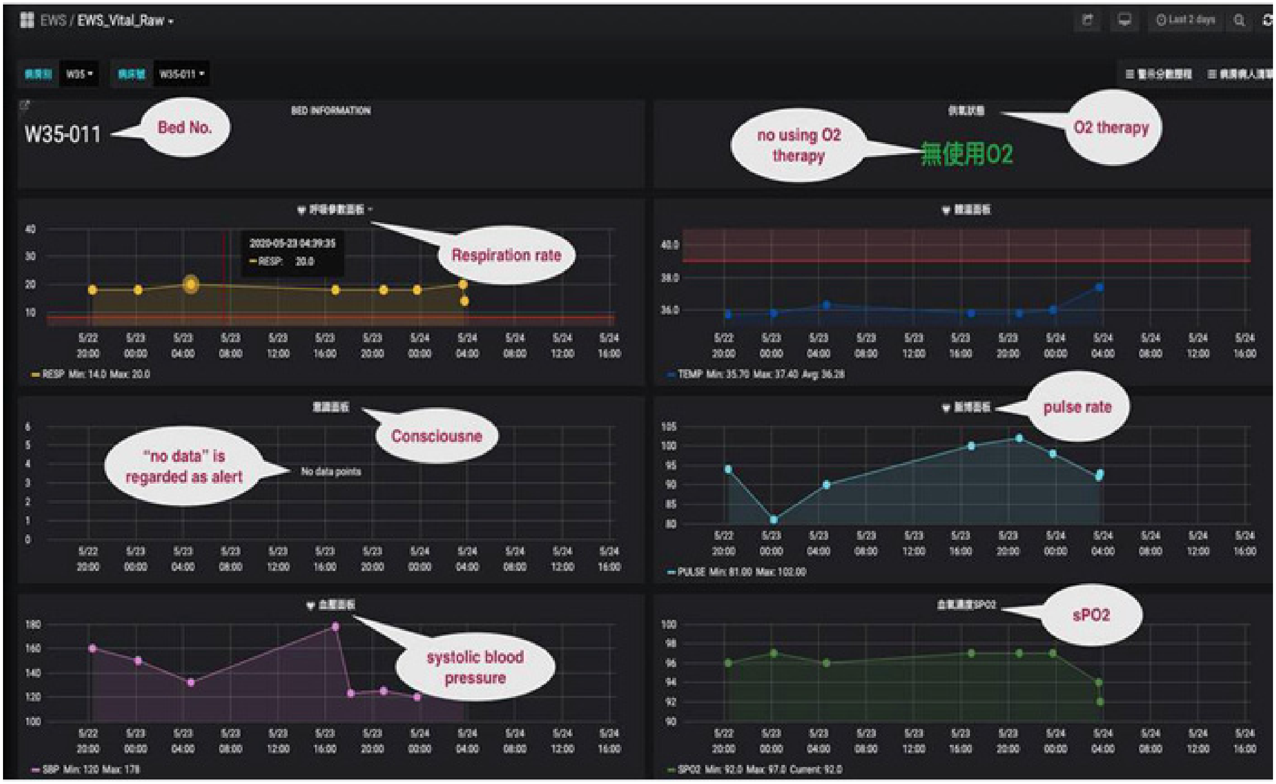

Supplementary Figure 2.The E-NEWS Dashboard was designed for each ward. The scores are shown in green (0~3), yellow (4~6) and red (≥7). We can trace the trend of the scores from the time the patient is admitted. The trend within the last 48 hours is set as the default. The E-NEWS dashboard for the division is the same as for the ward, except the list of divisions.

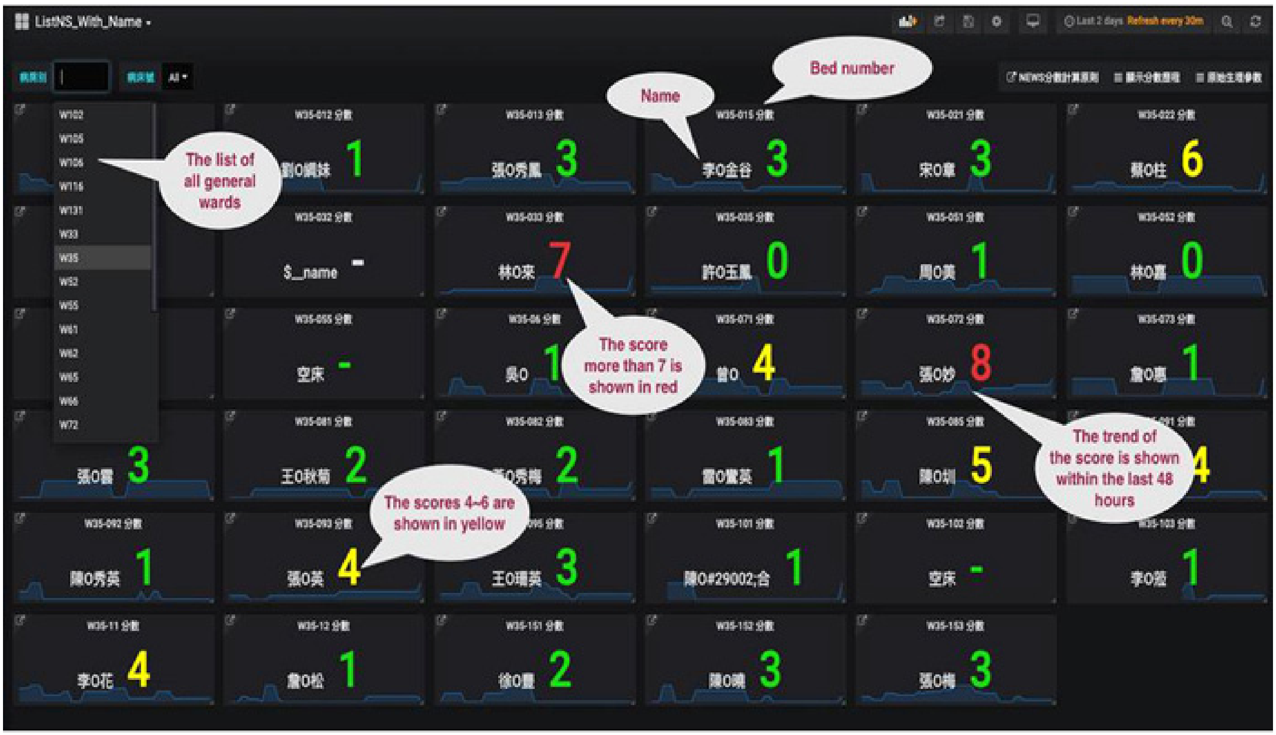

Supplementary Figure 3. If the E-NEWS meets the criteria, i.e., the E-NEWS increased to more than 4 in the last 12 hours and the last E-NEWS was ≥ 7, we sent a message to the care team (left) and to the in charge attending physician. (right).

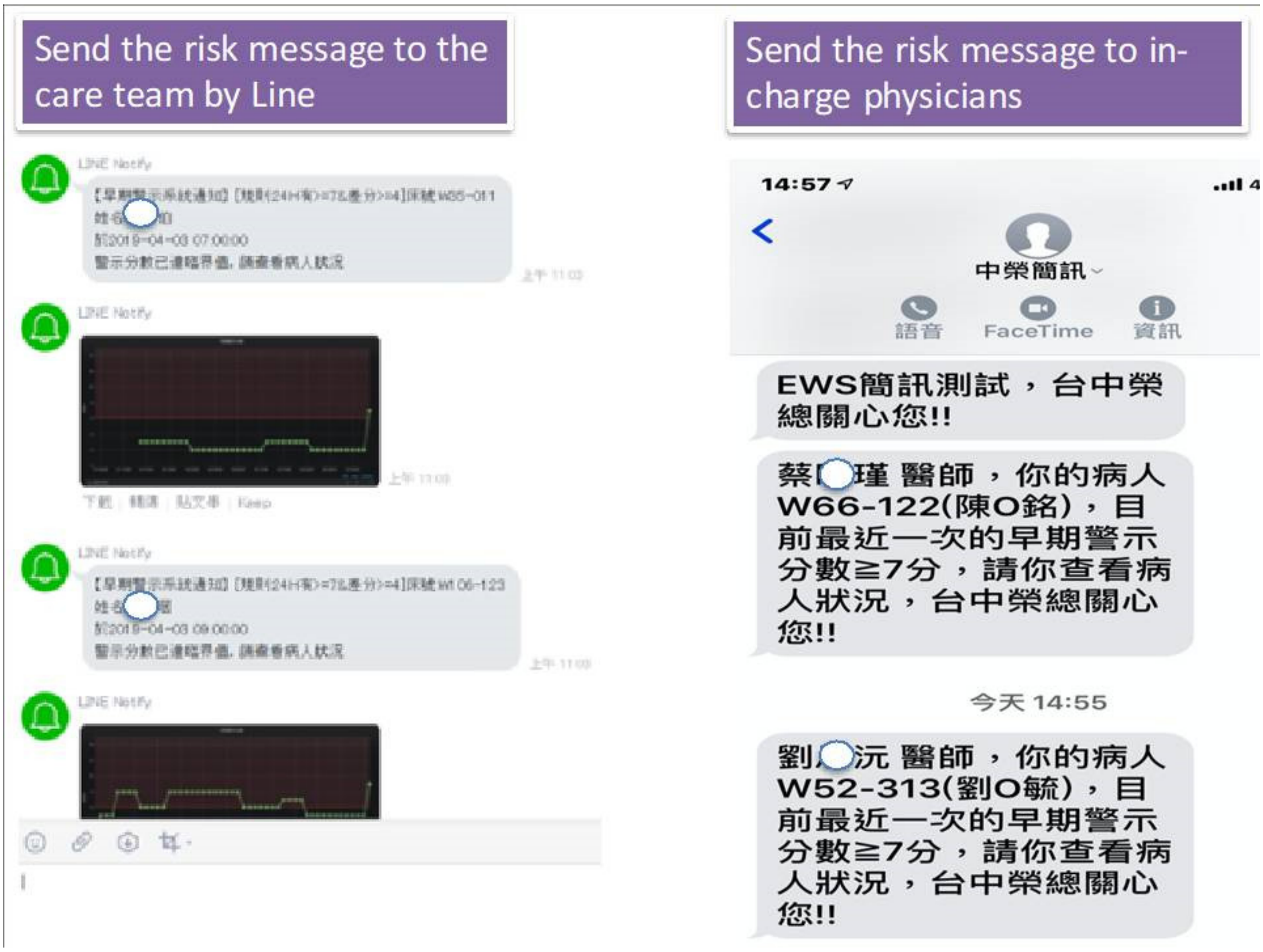

Supplementary Figure 4. This is the interface of the Nurse Information System (NIS) for the nurse shift. The score is transferred to the sheet of the shift automatically. Therefore, the score of each patient is checked every 8 hours.

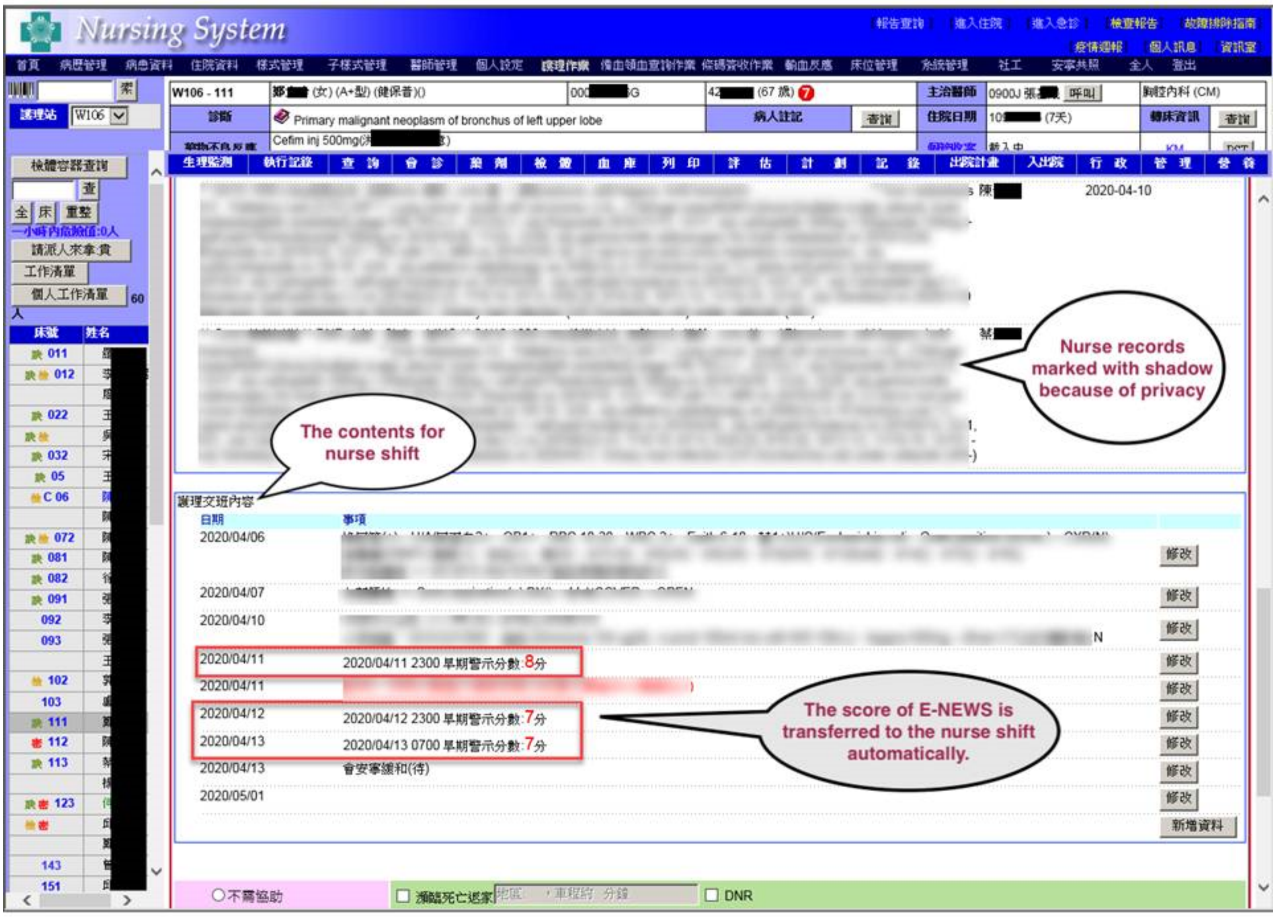

Supplementary data

We retrospectively reviewed those who fulfilled the criteria of E-NEWS scores increasing to more than 4 in the last 12 hours and the last E-NEWS ≥ 7 (that is, his in charge physician received alarm text message) in September of 2019. There were totally 496 patients impending deterioration. Sixty-seven patients (13.5%) died whereas 34 patients (6.9%) were transferred to intensive units in the following 48 hours. It is higher than that in general hospitalized patients. Abovementioned data justified that we used such criteria for sending alarm text message to in charge physician.

- Condition in the following 48 hours

	Patient No.	%
Stay in ward	395	79.6
Transfer to ICU	34	6.9
Died	67	13.5
Total	496	100.0

Supplementary Table 1. The ICD-9 and ICD-10 codes of co-morbidities

**Category**	**ICD-9**	**ICD-10**
**Cancer**	140–208	C01–C96, D03
**Cardiovascular disorder**	393–398, 401–405, 410–417, 420–429, 440–449	I05–13, I15, I20, I28, I30–I52, I70–I71, I75, I77, I97
**Neurological disorder (non-stroke)**	320–327, 330–337, 340–359	B45, E08–E13, E75, G00–G13, G20–G26, G30–G32, G35–G37, G40, G43, G44, G47, G50–G65, G70–G73, G80–G83, G90–G99
**Neurological disorder (stroke)**	430–438	G45, G46, I60–I69
**Respiratory disorder**	480–487, 490–496, 500–519	A22, A37, A48, B25, B44, J09–J18, J22, J39, J40–J45, J47, J60–J70, J80–J82, J84–J86, J90–J99, R09, R91
**Diabetes**	250	E08–E11, E13
**Gastroenterology**	530–537, 540–543, 550–553, 555–558, 560, 562, 564–579	B25, J86, K20–K23, K25–31, K35–K38, K40–K46, K50–K52, K55–K63, K63–K77, K80–K83, K85–K87, K90–K92, K94, R16, R18
**Renal disorder**	430–404, 582–583, 585–586, 588, V42, V45	I12–13, N03, N05–N08, N14–N19, Z94, Z99
